# Health facility readiness to provide integrated Family Planning, Maternal and Child Health (FPMCH) services in Nepal: Evidence from the comprehensive health facility survey

**DOI:** 10.1371/journal.pone.0264417

**Published:** 2022-02-25

**Authors:** Kiran Acharya, Dipak Subedi, Pawan Acharya

**Affiliations:** 1 New ERA, Rudramati Marga, Kalopul, Kathmandu, Nepal; 2 Ministry of Health and Population, Kathmandu, Nepal; 3 Department of Biostatistics and Epidemiology, Hudson College of Public Health, The University of Oklahoma Health Sciences Center, Oklahoma City, OK, United States of America; Vital Strategies, UNITED STATES

## Abstract

**Introduction:**

This study aimed to build the emergent body of evidence of family planning and maternal and child health (FPMCH) service integration benefits that can be useful in reaching the target of sustainable development goals (SDGs).

**Methods:**

We utilized data from the 2015 Nepal Health Facility Survey and used the World Health Organization service readiness manual for defining the service readiness indicator score of all services related to FPMCH integration. Composite readiness index for all services including family planning, antenatal care service, delivery and newborn service readiness index, and curative child care service readiness index was considered for the integration of all services (i.e. readiness for FPMCH). Multivariable linear regression models were fitted to examine the association between covariates and readiness score to provide integrated services.

**Results:**

The mean readiness score of integrated services in health facilities in Nepal was 52.1%. The services in private hospitals and peripheral health facilities had significantly lower readiness scores of integrated services compared to government hospitals. Compared to Province 3(Province 3 holds the national capital), Province 2 had significantly lower and Province 7 had significantly higher readiness scores. There is a marginally significantly higher readiness score of integrated services in the facilities where management meetings are held compared to where management meetings are never conducted. Interestingly, health facilities where external supervision occurred in the last 4 months had better service readiness of integrated services compared with those facilities with no external supervision. Similarly, the facilities that performed the routine quality assurance activities and facilities having a system for collecting opinion and being reviewed had a higher integrated services readiness score than their counterparts.

**Conclusion:**

This study found a scope of improvement in management practices in the health sector of Nepal especially for supportive supervision, quality assurance (QA) activities, monthly management meetings, and a system of collecting and reviewing opinions from the clients. Strengthening management practices especially, promoting supportive supervision and adhering to QA protocols may improve HF readiness to implement integrated FPMCH in Nepal. Being low readiness, there is an urgent need for policy reform to improve the integrated service readiness, particularly in Province 2.

## Introduction

The Millennium Development Goal (MDG) era saw vivid improvements in health goals for improving maternal and child health with a 44% reduction of maternal mortality ratio and about 22% of whom are in South Asian countries [1, 2]. Nepal has also proven a substantial decrease of maternal and child health mortality in two decades [2–4] while the use of the modern contraceptives method of family planning has been stagnant since the last decade [4].

The sustainable development goal (SDG), Goal 3- good health and wellbeing focuses most explicitly on health and covers key targets (SDG 3.7) to ensure universal access to sexual and reproductive health-care services, including family planning (FP), information, and education, and the integration of reproductive health into national strategies and programs [5]. MDG’s key lesson also was that the new health goal and related targets cannot be achieved without strengthening health systems broadly and linking the health sector with other sectors that address the structural determinants of health. Although Nepal’s Constitution 2015 guaranteed basic health care as a fundamental human right, access to high-quality care remains a privilege [6, 7]. Starting with Nepal’s National Health Policy 1991, the importance of multi-sectoral coordination, decentralized planning and management, and overall health system strengthening has been repeatedly articulated; however, the country’s aspiration for a robust health system has been unsatisfactorily realized [8]. What hinders progress towards people-centeredness in Nepal’s health system is the continued prioritization of disease-centered vertical programs for defining health and health system functions [9].

Nepal’s health sector strategy(2015–2020) is assembled on four strategic principles: equitable access to health services, quality health services, health systems reform, and multi-sectoral approach [10]. This was the core theme of the national health sector strategies under the focused area of Nepal’s community based primary health care as the foundation of universal health coverage (UHC) [11–13]. Family planning with maternal and child health service delivery integration shows potential in improving various outcomes [14]. However, significant evidence gaps remain. Rigorous research comparing outcomes of integrated with non‐integrated services, including cost, mortality and pregnancy‐related outcomes, is greatly needed to inform programs and policy [15]. Furthermore, the World Health Organization (WHO) has defined integrated health services as the management and delivery of health services as a result that users obtain a variety of preventive and curative services according to their necessities [16]. Integration also makes use of different service entry points, increases structural harmony, enables efficiencies, and builds a wider cross-cutting approach to deliver comprehensive care for clients with multiple health needs [16, 17]. Globally, the importance of integrating maternal, neonatal, child health, and nutrition (MNCHN) services, with family planning services is well accepted as a vital approach to improve maternal and child health and survival [18].

Family planning and maternal and child health (FPMCH) services in this study include any modern family planning service, antenatal care, delivery, and newborn care, and child curative care. The results of the secondary analysis will help in developing the evidence of FPMCH service integration benefits to describe lessons learned and evidence-informed applications from the evidence that can be useful in reaching the target of SDGs. These services of reproductive health are seen as an example of a wider podium that is not disease-specific but focuses more on a person-centric, holistic approach to health and well-being that could contribute to the broader discourse on the opportunities and known challenges for integrating health services.

Being a significant proportion of the total health services included in the primary health care system in Nepal, the availability of quality FPMCH determines people’s perception towards the overall health care delivery system. Nepal’s transformation from a centrally administered kingdom to a federal structure also demands a radical transformation in the country’s primary health care delivery system. Despite being a priority program, there is a gap in the evidence on the health sector’s ability and readiness to provide FPMCH services. Evaluation of nationally representative data to assess the health sector’s readiness to provide FPMCH services will be useful for managers to identify the current situation at the aggregate as well as provincial level and help them intervene at the weaker area in order to improve the service delivery system.

Under this circumstance, this study aimed to harness the publicly available nationally representative Nepal Health Facility Survey (NHFS) 2015 data to assess the integrated service’s readiness of FPMCH that are vital components of health facilities’ capacity to provide quality maternal and newborn care. The lessons learned both within and beyond the health sector to the wider agenda of linking policies and programs on FPMCH will inform future strategies towards a multi-sectoral developmental approach to attain the SDGs.

## Materials and methods

### Data source

This study is based on the publicly available secondary data analysis. We used data from the 2015 NHFS identical to the worldwide service provision assessment (SPA) conducted through the demographic and health survey (DHS) program. This survey is the first comprehensive facility-based survey conducted in Nepal, and the report is published elsewhere [19]. It combines the components of the United States Agency for International Development (USAID)-supported SPA of the DHS program, WHO’s Service Availability and Readiness Assessment, The United Nations Population Fund (UNFPA) sponsored Facility Assessment for Reproductive Health Commodities and Services and the Nepal-specific Service Tracking Survey. To provide a comprehensive picture of the strengths and weaknesses of the service delivery environment for each assessed service, the 2015 NHFS collected information from formal sector health facilities managed by the government and other authorities.

### Sample size and sampling procedure

The details of the survey design and sampling procedure is given in the survey final report [19]. In summary, a master list of 4,719 formal-sector health facilities in Nepal was obtained from the ministry of health and population (MoHP) and used as the sampling frame for the survey, a total of 1,000 facilities were selected for the survey. By design, the sample included all non -specialized government hospitals, all private hospitals with 100 or more inpatient beds, and all Primary Health Care Centers (PHCCs). Other facilities like Health Posts (HP), private hospitals with at least 15 beds but fewer than 100 beds, stand-alone HIV Testing and Counselling (HTC) sites, and Urban Health Clinics (UHCs) were selected randomly. Eight sampled facilities turned out to be duplicated, resulting in an effective sample size of 992 facilities. Of the 992 selected facilities, 29 either refused to participate in the survey or were not functional. Finally, a total of 963 facilities took part in the survey. However, we have excluded stand-alone HTCs (n = 23) standalone from the analysis because they only provide the HIV/AIDS-related services. Hence, the secondary data from 940 facilities were included in this study.

### Data collection and management

This study used publicly available data from NFHS 2015. The survey was conducted by the New ERA under the auspices of MoHP with technical assistance from ICF through its MEASURE DHS project. The survey used five types of data collection tools: facility inventory questionnaire; health provider interview questionnaire; observation protocols for antenatal care (ANC), FP, and services for sick children; and exit interview questionnaires for ANC and FP clients, postpartum mother, and for caregivers of sick children whose consultations were observed. Along with this country-specific health facility operational management committee (HFOMC) questionnaire was also designed for this survey. The details of the questionnaire can be found in the published report [19].

Most of the secondary data analyzed in this study were obtained from the facility inventory file, but one variable regarding staff who had received training in the last 24 months was obtained from the Health Provider file. The data in these files were edited, cleaned, and re-coding was performed for variables of interest to obtain meaningful information for addressing our research questions. The DHS program (www.dhsprogram.com.np) granted permission to access and utilize the 2015 Nepal Health Facility Survey datasets.

### Measurement of variables

In our study, facility readiness of FPMCH is defined as the willingness or state of the health facility to provide FP, ANC, delivery, and newborn care (basic obstetric and newborn care) and curative child care. Similarly, health provider training is defined as on-the-job training provided to health providers if they received any “in-service” training, including any training “updates” or “refresher” training within the past 24 months, without taking into consideration their roles to update their knowledge, skills, and technical competence to improve health care related to the services offered to clients. The facility readiness of FPMCH was measured based on the collective scores of FP, ANC, delivery and newborn care, and curative child care readiness indices. The scores and the readiness indicators were identified according to the WHO Service Availability and Readiness Assessment (SARA) Manual [20]. Using this manual, the FPMCH service readiness index was categorized up to four domains:staff and guidelines, equipment, diagnostic, and medicines/commodities. The different tracer items on the specific domain of the FPMCH is given in S1 Table.

Each index (FP, ANC, delivery, and newborn care and curative child care service readiness) was then totaled by adding the presence of each indicator with equal weight given to each of the domains and each of the indicators within the domains [21–24]. As the target was 100%, each domain (for family planning and delivery and newborn care) accounted for 33.3% (100%/3) of the index while for the ANC and curative child care services, each domain accounted for 25% (100%/4). The percentage for each indicator within the domain was equal to 33.3% for family planning and delivery and newborn care and 25% for ANC and curative child care services divided by the number of indicators in that domain. The FPMCH service readiness indices for each facility were then calculated by summing the percentages of all domains making the scores to a 0–100%. The summary of the measurement procedure of the readiness score can be seen in S2 Table.

### Independent variables

Facility type was categorized as government hospitals (zonal and above hospitals and district level hospitals), private hospitals, and peripheral health facilities (PHCCs, HPs, and UHCs). Managing authority was categorized as public or private. Similarly, other geographical and management related covariates for service readiness are categorized as; Ecological region into mountain, hill, and terai. Province 2 has since changed its name to Madhes Province (January, 2022), Province 3 to Bagmati Province (January, 2020), Province 4 to Gandaki Province (July 2018), Province 5 to Lumbini Province (October 2020), Province 6 to Karnali Province (February 2018), Province 7 to Sudurpashchim Province (September 2018). The remaining one province have not adopted permanent names as of the time of this publication. Provinces were categorized into Province 1 to Province 7, since these are the province names that were in effect at the time of the survey. Routine management meetings were categorized as “Yes” for facilities reporting that they performed routine management meetings at least quarterly and “No” for those that reported having no such meetings at least every quarter. External supervision was coded as “Yes” for facilities reporting receiving external supervision in the past 3 months and “No” for those without such supervision. Similarly, routine quality assurance activity was coded as “performed” for facilities reporting that it routinely carries out quality assurance activities and had documentation of a recent quality assurance activity and “Not performed” for those without such quality assurance activities. Similarly, the system of collecting and reviewing the opinions was coded as “Reviewed” for facilities reporting there is the system of collecting and reviewing client’s opinions and “Not reviewed” for those not having such a system. This could be a report or minutes of a quality assurance meeting, a supervisory checklist, a mortality review, or an audit of records or registers. For the location of the facilities (rural and urban), it was not available in the dataset but we classified them according to the current administrative units of each facility’s available global positioning system (GPS) location [24] and defined the rural areas (rural municipality) and urban areas (metropolitan/sub-metropolitan city and municipality) [25].

### Statistical analysis

In descriptive analyses, categorical variables were summarized using frequency count and proportions with 95%CI of the proportion. A box plot was created to visualize the readiness score among the health facilities for individual programs. We used bivariate and multivariable linear regression analyses to assess the relationship between covariates and an outcome variable (Facility readiness to provide FPMCH services or integrated services). Model validation and assessment of assumption of linear regression model was performed using residual plots (Not shown here). We assessed the linearity in the association between dependent and independent variables, statistical independence of the residuals, constant variance of the errors, and normality of the error distribution using graphical methods. The assumptions were satisfied in the univariate and final multivariable linear regression models. We checked the interaction among independent variables in the regression model and detected no statistically significant interaction. Unadjusted, adjusted coefficients, standard error and p value is shown in the model and a p-value <0.05 was considered statistically significant throughout the analysis. Statistical analysis was performed in STATA 15.0 accounting for the sample weight and survey design.

## Ethical approval

The 2015 NHFS survey was approved by the Nepal Health Research Council (NHRC), and the Institutional Review Board of ICF in the USA. Before interviews were performed, informed consent was obtained from the participants present at the facility. The respondents were adequately informed regarding all relevant aspects of the study, including its aim and interview procedures. Respondents that accepted their facilities to participate in the study signed informed consent. Measure DHS granted permission to use the data for this analysis.

## Results

### Background characteristics of selected health facilities

The majority of the facilities (90%) were peripheral health facilities and 93% were public facilities. The majority (922, 77.6%) reported having routine management meetings. About 51% of the facilities lie in the hill region followed by terai (36%) and mountain (13%). About two third (66%) of health facilities had regular monthly management meetings and had received external supervision (63%) in the last 4 months of the survey assessment. Only one fifth of the facilities reported that they had performed routine quality assurance activities and fewer than 10 facilities had a system of collecting opinions and were reviewed (Table 1).

**Table 1:** Percentage distribution of surveyed facilities (excluding standalone HTCs) according to background characteristics (n = 940).

Variable	Number (weighted)	%
**Facility type**		
Government hospitals	21	2.3
Private hospitals	70	7.4
Peripheral health facilities	849	90.3
**Managing authority**		
Public	870	92.6
Private	70	7.4
**Location**		
Rural	462	49.1
Urban	478	50.9
**Ecological region**		
Mountain	118	12.6
Hill	482	51.2
Terai	340	36.2
**Province**		
Province 1	164	17.4
Province 2	171	18.2
Province 3	185	19.7
Province 4	119	12.7
Province 5	137	14.7
Province 6	74	7.9
Province 7	89	9.5
**Monthly management meetings**		
Never	183	19.5
Sometimes	133	14.2
Regularly	624	66.4
**External supervision in last 4 months**		
Not done	350	37.3
Done	590	62.8
**Routine quality assurance (QA) activities**		
Not performed	752	80.0
Performed	188	20.0
**System of collecting opinion and reviewed**		
Not reviewed	862	91.7
Reviewed	78	8.3

### Availability of services and health facility readiness for offering FPMCH services

Table 2 presents the distributions of availability and readiness of health facilities to provide FPMCH services. Almost all of the health facilities included in the study reported the availability of child curative care, antenatal care, and family planning services while only about half (49%) of the facilities reported the availability of delivery and newborn care services. Based on the WHO SARA readiness indicator score, 68% of health facilities were ready to offer FP whereas about 50% of facilities were ready to offer child curative care and ANC but only 28% of facilities were ready to offer delivery and newborn care services. Among the health facilities considered in the analysis, the mean readiness score of the facilities to offer integrated services was 52.1% (Table 2).

**Table 2:** Percentage distribution of health facilities with the availability and readiness to family planning, maternal and child health services (n = 940).

Variable	Number (weighted)	% (95% CI)
**Service availability**		
Family planning	919	97.7 (96.9–98.3)
Antenatal care	919	97.8 (96.1–98.8)
Delivery and newborn care	457	48.7 (44.6–52.7)
Child curative care	934	99.4 (98.7–99.7)
All service	445	47.3 (43.3–51.4)
**Components of FP readiness index**		
Staff and guidelines	203	21.6 (19.0–24.1)
Equipment	795	84.6 (81.4–87.9)
Medicine/commodities	909	96.7 (95.9–97.6)
Overall FP readiness index	635	67.6 (66.1–69.1)
**Components of ANC readiness index**		
Staff and guidelines	239	25.4 (22.8–27.9)
Equipment	790	84.0 (80.6–87.5)
Diagnostics	147	15.6 (14.1–17.1)
Medicine/commodities	667	71.0 (69.5–72.6)
Overall ANC readiness index	461	49.0 (47.7–50.4)
**Components of delivery and newborn care readiness index**		
Staff and guidelines	133	14.1 (12.0–16.2)
Equipment	371	39.5 (37.0–42.0)
Medicine/commodities	290	30.8 (28.6–32.9)
Overall delivery and newborn care readiness index	264	28.1 (26.1–30.1)
**Components of child curative care readiness index**		
Staff and guidelines	387	41.2 (38.3–44.1)
Equipment	601	63.9 (62.4–65.4)
Diagnostics	151	16.1 (14.5–17.7)
Medicine/commodities	714	76.0 (74.6–77.3)
Overall child curative care readiness index	463	49.3 (48.2–50.4)
**Overall FPMCH integration readiness index**	**490**	**52.1 (51.1–53.0)**

**Fig 1:**
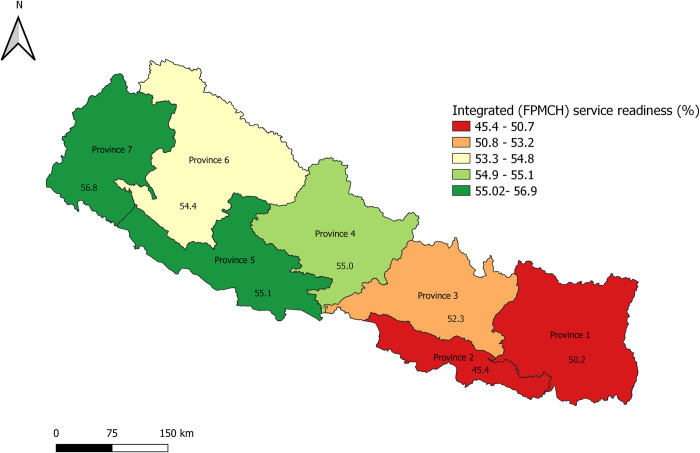
Mean service readiness score for integrated FPMCH services according to province in Nepal.

According to the provinces, Province 2 had the lowest and Province 7 had the highest proportion of health facilities with the readiness to provide integrated FPMCH services. Fig 1 shows the health facility readiness status according to the provincial level administration (Fig 1).

Health facilities readiness score for specific services according to the provincial level administration is shown in S1 Appendix.

**Fig 2:**
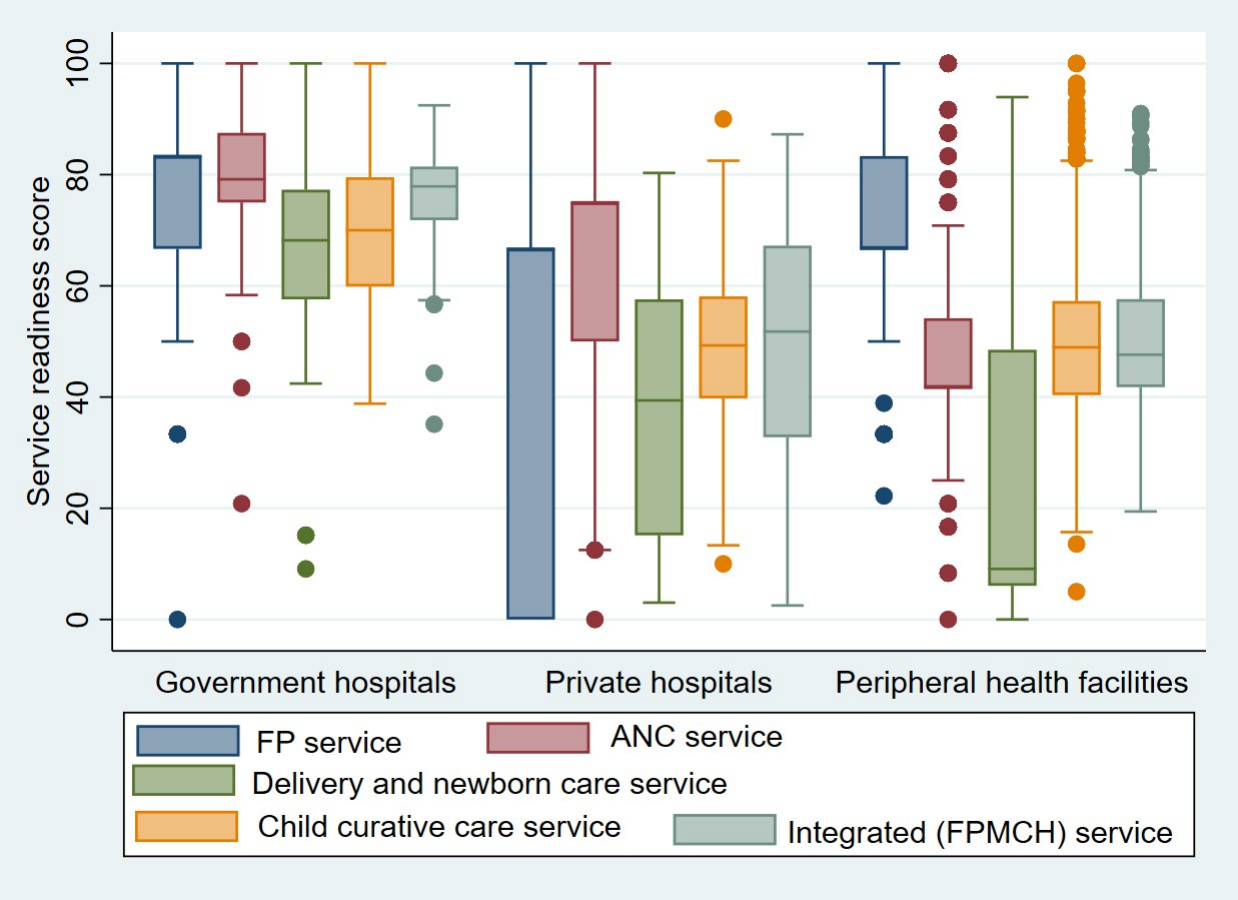
Service readiness score for specific and integrated FPMCH services according to the type of facility.

Fig 2 shows a significant difference in the specific service and integrated readiness score for FPMCH services among different types of health facilities.

### Determinants of facility readiness for the integration of FPMCH services

The multivariable linear regression analysis showed that the services in private hospitals and peripheral health facilities had significantly lower readiness scores (-23.58% and -22.35% points lower respectively) of integrated services compared to government hospitals. Province 2 had lower readiness (4.78% points lower), and Province 7 had significantly higher readiness (5.62% points higher) of the integrated services compared to Province 3 (Province 3 holds national capital). There is marginally significantly higher readiness score of integrated services in the facilities where management meetings are held (sometimes:3.21% points higher and regularly:2.98% points higher) compared to where management meetings are never conducted. Interestingly, health facilities where external supervision occurred in the last 4 months had better service readiness of integrated services compared with those facilities with no external supervision (4.0% points better than those not occurring). Similarly, the facilities that performed the routine quality assurance activities had a higher integrated services readiness score (2.02% points better than those facilities not performed). Similarly, the integrated service had better readiness at facilities having the system of collecting and reviewing opinion (4.71% points better than its counterpart) (Table 3).

**Table 3:** Determinants of facility readiness integration for the FPMCH service in bivariate and multivariable linear regression (n = 940).

Variable	Unadjusted		Adjusted	
	Coefficient	Standard error	Coefficient	Standard error
**Facility type**				
Government hospitals	ref		ref	
Private hospitals	-24.52***	2.37	-23.58***	2.47
Peripheral health facilities	-25.25***	1.51	-22.35***	1.57
**Location**				
Rural	ref			
Urban	1.35	1.01	1.16	0.99
**Ecological region**				
Mountain	ref		ref	
Hill	-0.96	1.16	0.68	1.14
Terai	-2.76*	1.34	0.002	1,72
**Province**				
Province 3	ref		ref	
Province 1	-2.08	1.53	-0.76	1.56
Province 2	-6.89***	1.61	-5.78**	2.09
Province 4	2.67	1.68	2.38	1.60
Province 5	2.77	1.59	2.75	1.77
Province 6	2.13	1.56	3.09	1.65
Province 7	4.53**	1.74	4.86**	1.67
**Regular management meetings**				
Never	ref		ref	
Sometimes	4.10*	1.67	3.21*	1.49
Regularly	4.49**	1.34	2.98*	1.18
**External supervision in last 4 months**				
Not done	ref.			
Done	5.33***	1.02	4.00***	0.94
**Routine quality assurance (QA) activities**				
Not performed	ref.		ref.	
Performed	3.53**	1.13	2.02*	1.06
**System of collecting opinion and reviewed**				
Not reviewed	ref.		ref.	
Reviewed	6.82**	2.01	4.71*	2.12

*p<0.05

**p<0.01

***p<0.001

## Discussion

To our knowledge, this is the first study to assess the readiness of health facilities and factors associated to provide integrated FPMCH services at a national scale in Nepal. This study reports that regular monthly meetings did not happen in among one fifth of the health facilities. Also, an overwhelming majority (80%) of the facilities did not perform routine QA activities. Among the services, delivery and newborn care were available only among one in every two facilities i.e 49%, and about 47% of facilities had availability of all the (FP, ANC, Delivery and newborn care, and child curative care) services. Based on the WHO SARA manual [26], the mean of 52.1% of the facilities was ready to provide FPMCH services (lowest 28.1% in delivery and newborn care to highest 71% in ANC). Furthermore, this study found facility type, provincial location, managerial practices including external supervision, routine QA activities, and system of collecting and reviewing client opinion were significantly associated with the health facility readiness to provide integrated FPMCH in Nepal.

Compared to government hospitals, private hospitals, and peripheral health facilities had significantly lower readiness scores to provide integrated services. This is consistent with the similar studies conducted in Nepal [24, 27]. This result may represent the limited availability of equipment, medicines of delivery, and newborn care services at peripheral health facilities and also the limited availability of FP methods and priority medicines of MCH in private hospitals [19]. Recently, the health sector has made a significant investment in government hospitals to enable them to provide basic and comprehensive reproductive health services [28, 29]. The services provided in the government hospitals are of a wider-range and the listed FPMCH services are provided free of charge to the users [30]. Peripheral health facilities also provide services free of charge but they often undergo several limitations related to human resource, financial, and logistic issues [31].

Interestingly, health facilities from Province 2 were significantly lower, but Province 7 had a significantly higher score of readiness compared to the Province 3 (Province 3 holds the national capital). Data from the health management information system (HMIS) indicate most of the maternal health and family planning-related indicators of Province 7 are better compared to Province 3 or the national average [32]. Better management of the health program, commitment, and dedication from leadership and health workers might be the factor influencing higher readiness in Province 7 compared to province 3. Qualitative exploratory studies will be helpful to identify the factors contributing to the higher readiness among the facilities of province 7. On the other hand, the low readiness of integrated services in Province 2 is similar to the study done by Acharya et al that showed the mean general health service readiness score was lowest in Province 2 [22]. Though plain area the Province 2 had also lower socio-economic status compared to the Province 3 as well as with other provinces [33, 34]. General underdeveloped nature of the Province 2 compared to Province 3 might have reflected in the lower readiness score among the facilities within the province.

The facilities conducting regular management meetings had higher readiness scores compared to the facilities with no regular meetings. This study also found facilities with external supervision in the last 4 months and routine quality assurance activities had significantly higher readiness compared to the facilities where those activities were not conducted. This is similar to other studies conducted in Nepal [22, 24]. Unlike in other developing countries, due to the primary health care system structure, the majority of the health services are directly managed by district health authorities in Nepal, and therefore, supervision generally involves the district-level health authority’s visit to primary care facilities in Nepal [31]. The supervision includes interaction with staff and motivation, record review, problem-solving, and observing service provision practices, often using a predetermined checklist [35]. The external supervision must be supportive of the supervisee rather than just the fault-finding process. Regular support to the health facility not just prepares the facility to provide the integrated services but also is an essential component of the successful integration of FP and MCH [36] and also other services [37]. These supportive activities provide opportunities for the supervisee staff to gain new insights to identify the challenges and address them and also share their feedback to higher authorities requesting support if needed. This is also an opportunity to review the status and progress of the health facility’s performance and develop contingency plans when required. Supportive supervision was found helpful in human resource management for integrated service delivery in Tanzania [38]. Frequent supervision was also found helpful in improving service delivery elsewhere [39].

Facilities with regular QA activities were found to have higher percentage points of readiness to provide integrated services compared to the facilities without regular QA activities. QA is a system that monitors the quality of provided services by detecting problems, and implementing change strategies to address those problems [19]. Supervision and quality assurance activities also help estimate any upcoming challenges and shortcomings. QA also involves reviewing and auditing of all the essential components such as human resource and guideline, equipment, diagnostic capacities and medicine, and essential commodities to provide the integrated services.

Similarly, the integrated service had better readiness at facilities having the system of collecting and reviewing opinion (4.71% points better than its counterpart). This could be because those facilities with the mechanism for collecting and reviewing opinion from the clients were able to receive feedback from their clients and were able to perform troubleshooting. It is also possible that those facilities had better organization and leadership pattern and a strong stakeholder ship among all concerned parties including health workers, community people and local leaders. Another study also reported higher readiness for general health services provision among the health facilities having the system of collecting and reviewing compared to others [22].

NHFS 2015 reported one out of 10 FP providers had ever received in-service training on long-active reversible contraceptive methods and only 16% reported they received in-service training related to FP in the 24 months before the survey [19]. In this low level of training, supportive supervision, and QA would play a key role in updating the knowledge, skills, and practices of the FP service providers. Lack of adequate skills and adequate training were reported as barriers to deliver integrated sexual and reproductive services elsewhere [37, 40, 41].

### Strengths and limitations

This study used readily available nationally representative data collected from internationally comparable survey methodology using the WHO SARA manual [26]; thus it does not represent the user’s perspectives of integrated care services. It should also be noted that all the elements of information important for building a complete picture of service integration were not collected in the survey instruments. We however sourced the 2015 NHFS survey for the current study recognizing its essential role that integrated FPMCH service delivery plays in ensuring universal health coverage and quality of care. So far, another round of such comprehensive health facility surveys has not been conducted; the evidence presented in this analysis should be interpreted as a baseline of integration in a country’s health system. Further, the survey is cross-sectional and the association presented in the findings should not be interpreted as causality.

### Context and public health significance

This study is the first to assess the evaluation of the health sector in terms of availability and readiness to provide integrated FP and MCH services in Nepal; therefore, the estimates calculated in this study will provide comparison data for future studies.

Family Planning Program has a long history in Nepal. It was started by the Family Planning Association of Nepal with support from the International Planned Parenthood Federation (IPPF) in 1959 [42]. Before 1998, service standards were fragmented and services were not consistently provided at designated health facility levels but the first National Reproductive Health Strategy in 1998 conceptualized an integrated approach and merged the previously vertical programs of family planning, safe motherhood, and child health [43]. Also, one of the objectives of the National Health Policy(NHP) of Nepal is to “establish an effective and accountable health care delivery system with essential drugs, medical equipment, technologies, and skilled human resources to ensure citizens’ access to quality health services” [19, 44]. The NHP also includes policies to deliver an effective, efficient, and equitable distribution of quality health care services throughout the nation. It also emphasizes partnership, management, and strengthening of the health sector.

Nepal’s National Reproductive Health Strategy is aligned with the policies adopted in the National Health Policy, Second Long-Term Health Plan, and Nepal Health Sector Program, which aims at reducing infant, child, and maternal morbidity and mortality, and reducing total fertility rate. The National Reproductive Health Strategy comprises the implementation of the integrated Reproductive Health Package at all levels of health institutions as well as at the community level based on standardized and clinical protocols and operational guidelines. Several national policies, programs, and strategies explicitly mentioned the integration of various reproductive health elements to increase the availability and utilization of services [10, 45–52].

Integrated health services from the service provider’s perspective refer to a variety of services for a target population provided at one institution and under one management system whereas from the user’s perspective is the opportunity to receive coordinated care rather than having separate appointments for separate interventions. Family planning is considered to be the entry point of the continuum of care. Simultaneous provision of family planning and maternal child care is cost-effective and also saves lives and time [53–55]. In Nepal, the MMR has come down to 258 (per 100,000 live births). Similarly, the Contraceptive Prevalence Rate (CPR) has increased to 47.1 percent, while the Total Fertility Rate (TFR) has decreased to 2.3. Furthermore, the adolescent fertility rate remains high at 71 live births per 1000 women aged 10–14. Major strategies that have been adapted to reduce risks during pregnancy and childbirth and address factors associated with mortality and morbidity include expansion of services along with increment in accessibility and utilization.

The integration of FP to MCH is considered a milestone in public health service delivery in Nepal. The evaluation report of integrated woman and reproductive health services in Nepal found integrated health services as justifiable, effective, efficient, impactful, and sustainable. Studies from other nations indicate that integrated FP/MCH services provide various opportunities to streamline and improve care for increasing women’s reproductive health and the health of their children. Integrated services also reach more clients by using all opportunities for service delivery, requiring fewer provider-client contacts [14, 56–58]. Strengthening management practices including regular meetings, supportive supervision, and timely addressing the implementation challenges experienced by the health facilities would help accelerate Nepal’s journey toward achieving SDG-3. Also, the finding of this study would provide a baseline estimate for future studies to assess health facility readiness to provide integrated FPMCH in Nepal.

## Conclusion

This study further analyzed national representative secondary data from NFHS 2015. This study found 52.1% of facilities across the nation were ready to provide integrated FPMCH services. Furthermore, there is a significant disparity across the provinces in Nepal. Further exploring the factors behind the success of Province 7 would help other provinces adopt contributing factors to higher readiness to provide integrated FPMCH. There was a positive correlation between facility type, regular external supervision, and routine QA activities, and the HF readiness to provide integrated services. A more decentralized approach to strengthening management practices especially, promoting supportive supervision and adhering to QA protocols might improve HF readiness to implement integrated family planning and maternal-child health services in Nepal.

## Supporting information

S1 Table Tracer items of each domain of FPMCH. (DOCX)

S2 Table Summary of the measurement procedure of FPMCH readiness score. (DOCX)

S1 Appendix Service readiness score (%) for specific services by province. (DOCX)

## Abbreviations

ANC: Antenatal care
aOR: Adjusted odds ratio
CI: Confidence Interval
DHS: Demographic and Health Survey
FP: Family planning
FPMCH: Family planning, maternal and child health
HP: Health post
HTC: HIV testing and counselling
MCH: Maternal and child health
MDG: Millennium development goal
MoHP: Ministry of Health and Population
NHFS: Nepal Health Facilities Survey
NHP: National health policy
NHRC: Nepal Health Research Council
PHCC: Primary health care centres
QA: Quality assurance
RH: Reproductive health
SARA: Service availability and readiness assessment
SDG: Sustainable development goals
SPA: Service Provision Assessment
UHC: Universal health coverage
UHCs: Urban health clinics
WHO: World health organization
