# Supplementary material for: Health facility readiness to provide integrated Family Planning, Maternal and Child Health (FPMCH) services in Nepal: Evidence from the comprehensive health facility survey
Source: PLoS One. 2022 Feb 25;17(2):e0264417. doi: 10.1371/journal.pone.0264417 (PMC8880709; doi:10.1371/journal.pone.0264417)
Supplement: S1 Table — (DOCX) [file pone.0264417.s001.docx]

S1 Table. Tracer items of each domain of FPMCH

| Indicators | Domain | | | |
| --- | --- | --- | --- | --- |
|  | Staff and guidelines | Equipment | Diagnostics | Medicines and commodities |
| FP | Guidelines on FP  Staff trained in FP | Blood pressure (BP) apparatus | Not Applicable | Combined estrogen progesterone oral contraceptive pills  Injectable contraceptives  Condoms |
| ANC | Guidelines on ANC  Staff trained in FP | Blood pressure (BP) apparatus | Haemoglobin (Hb)  Urine dipstick- protein | Iron and folic acid combined tablets  Tetanus diphtheria vaccine  Albendazole |
| Delivery& newborn care | Guidelines on delivery and new born care  Staff trained in delivery and newborn care | Emergency transport  Sterilization equipment  Examination light  Delivery pack  Suction apparatus (mucus extractor)  Manual vacuum extractor  Vacuum aspirator or D&C kit (with speculum)  Neonatal bag and mask  Delivery bed  Partograph  Gloves | Not Applicable | Injectable antibiotic  Injectable uterotonic  Injectable magnesium sulphate  Injectable diazepam  Intravenous fluids  Skin disinfectant  Antibiotic eye ointment  Chlorhexidine  Injectable gentamicin  Injectable ceftriaxone  Amoxicillin suspension |
| Child curative care | Guidelines on IMCI/IMNCI  Staff trained in IMCI/IMNCI | Child and infant scale  Length/height measuring equipment  Thermometer  Stethoscope  Growth chart | Haemoglobin (Hb)  Test parasite in stool (general microscopy)  Malaria diagnostic capacity | Oral rehydration solution (ORS)  Amoxicillin  Cotrimoxazole  Paracetamol  Vitamin A  Albendazole  Zinc Sulphate |
