# Supplementary material for: Health facility readiness to provide integrated Family Planning, Maternal and Child Health (FPMCH) services in Nepal: Evidence from the comprehensive health facility survey
Source: PLoS One. 2022 Feb 25;17(2):e0264417. doi: 10.1371/journal.pone.0264417 (PMC8880709; doi:10.1371/journal.pone.0264417)
Supplement: S2 Table — (DOCX) [file pone.0264417.s002.docx]

**Supplementary table 2 Summary of measurement procedure of FPMCH readiness scores**

| **Domain** | **Indicators (Tracer items)** | **Measurement** | **Percent score (%)** | |
| --- | --- | --- | --- | --- |
|  |  |  | **Indicator** | **Domain** |
| **FP service readiness index** | | | | |
| Staff and guidelines | Guidelines for diagnosis and treatment of FP | Yes  No | 16.67  0.00 | 33.33 |
|  | At least one staff member trained in FP | Yes  No | 16.67  0.00 |  |
| Equipment | Blood pressure (BP) apparatus | Yes  No | 33.33  0.00 | 33.33 |
| Medicines and commodities | Combined estrogen progesterone  oral contraceptive pills | Yes  No | 11.11  0.00 | 33.33 |
|  | Injectable contraceptives | Yes  No | 11.11  0.00 |  |
|  | Condoms | Yes  No | 11.11  0.00 |  |
| **Total FP readiness index score** | | | | 100.00 |
| **ANC service readiness index** | | | | |
| Staff and guidelines | Guidelines for diagnosis and treatment of ANC | Yes  No | 12.5  0.00 | 25.00 |
|  | At least one staff member trained in ANC | Yes  No | 12.5  0.00 |  |
| Equipment | Blood pressure (BP) apparatus | Yes  No | 25.00  0.00 | 25.00 |
| Diagnostics | Hemoglobin (Hb) | Yes  No | 12.5  0.00 | 25.00 |
|  | Urine dipstick- protein | Yes  No | 12.5  0.00 |  |
| Medicines and commodities | Iron and folic acid combined tablets | Yes  No | 8.33  0.00 | 25.00 |
|  | Tetanus diphtheria vaccine | Yes  No | 8.33  0.00 |  |
|  | Albendazole | Yes  No | 8.33  0.00 |  |
| **Total ANC readiness index score** | | | | 100.00 |
| **Delivery and new born care index** | | | | |
| Staff and guidelines | Guidelines for delivery and newborn care | Yes  No | 16.67  0.00 | 33.33 |
|  | Staff trained in delivery and newborn care | Yes  No | 16.67  0.00 |  |
| Equipment | Emergency transport | Yes  No | 3.03  0.00 | 33.33 |
|  | Sterilization equipment | Yes  No | 3.03  0.00 |  |
|  | Examination light | Yes  No | 3.03  0.00 |  |
|  | Delivery pack | Yes  No | 3.03  0.00 |  |
|  | Suction apparatus (mucus extractor) | Yes  No | 3.03  0.00 |  |
|  | Manual vacuum extractor | Yes  No | 3.03  0.00 |  |
|  | Vacuum aspirator or D&C kit (with speculum) | Yes  No | 3.03  0.00 |  |
|  | Neonatal bag and mask | Yes  No | 3.03  0.00 |  |
|  | Delivery bed | Yes  No | 3.03  0.00 |  |
|  | Partograph | Yes  No | 3.03  0.00 |  |
|  | Gloves | Yes  No | 3.03  0.00 |  |
| Medicines and commodities | Injectable antibiotic | Yes  No | 3.03  0.00 | 33.33 |
|  | Injectable uterotonic | Yes  No | 3.03  0.00 |  |
|  | Injectable magnesium sulphate | Yes  No | 3.03  0.00 |  |
|  | Injectable diazepam | Yes  No | 3.03  0.00 |  |
|  | Intravenous fluids | Yes  No | 3.03  0.00 |  |
|  | Skin disinfectant | Yes  No | 3.03  0.00 |  |
|  | Antibiotic eye ointment | Yes  No | 3.03  0.00 |  |
|  | Chlorhexidine | Yes  No | 3.03  0.00 |  |
|  | Injectable gentamicin | Yes  No | 3.03  0.00 |  |
|  | Injectable ceftriaxone | Yes  No | 3.03  0.00 |  |
|  | Amoxicillin suspension | Yes  No | 3.03  0.00 |  |
| **Total Delivery and newborn care index score** | | | | 100.00 |
| **Delivery and new born care index** | | | | |
| Staff and guidelines | Guidelines for curative services for sick children | Yes  No | 12.50  0.00 | 25.00 |
|  | Staff trained in delivery and newborn care | Yes  No | 12.50  0.00 |  |
| Equipment | Child and infant scale | Yes  No | 5.00  0.00 | 25.00 |
|  | Length/height measuring equipment | Yes  No | 5.00  0.00 |  |
|  | Thermometer | Yes  No | 5.00  0.00 |  |
|  | Stethoscope | Yes  No | 5.00  0.00 |  |
|  | Growth chart | Yes  No | 5.00  0.00 |  |
| Diagnostics | Hemoglobin (Hb) | Yes  No | 8.33  0.00 | 25.00 |
|  | Test parasite in stool (general microscopy) | Yes  No | 8.33  0.00 |  |
|  | Malaria diagnostic capacity | Yes  No | 8.33  0.00 |  |
| Medicines and commodities | Oral rehydration solution (ORS) | Yes  No | 4.17  0.00 | 25.00 |
|  | Amoxicillin | Yes  No | 4.17  0.00 |  |
|  | Cotrimoxazole | Yes  No | 4.17  0.00 |  |
|  | Paracetamol | Yes  No | 4.17  0.00 |  |
|  | Vitamin A | Yes  No | 4.17  0.00 |  |
|  | Albendazole | Yes  No | 4.17  0.00 |  |
|  | Zinc Sulphate | Yes  No | 4.17  0.00 |  |
| **Total curative services for sick children index score** | | | | 100.00 |
|  | | | | |
| **FPMCH readiness index** | | | | |
| **FP** | Staff and guidelines | Yes  No | 8.33  0.00 | 25.00 |
|  | Equipment | Yes  No | 8.33  0.00 |  |
|  | Medicines and commodities | Yes  No | 8.33  0.00 |  |
| **ANC** | Staff and guidelines | Yes  No | 6.25  0.00 | 25.00 |
|  | Equipment | Yes  No | 6.25  0.00 |  |
|  | Diagnostics | Yes  No | 6.25  0.00 |  |
|  | Medicines and commodities | Yes  No | 6.25  0.00 |  |
| **Delivery and newborn care** | Staff and guidelines | Yes  No | 8.33  0.00 | 25.00 |
|  | Equipment | Yes  No | 8.33  0.00 |  |
|  | Medicines and commodities | Yes  No | 8.33  0.00 |  |
| **Curative services for sick children** | Staff and guidelines | Yes  No | 6.25  0.00 | 25.00 |
|  | Equipment | Yes  No | 6.25  0.00 |  |
|  | Diagnostics | Yes  No | 6.25  0.00 |  |
|  | Medicines and commodities | Yes  No | 6.25  0.00 |  |
| **Total FPMCH index score** | | | | 100.00 |
