# Supplementary figures and images for: Health facility readiness to provide integrated Family Planning, Maternal and Child Health (FPMCH) services in Nepal: Evidence from the comprehensive health facility survey
Source: PLoS One. 2022 Feb 25;17(2):e0264417. doi: 10.1371/journal.pone.0264417 (PMC8880709; doi:10.1371/journal.pone.0264417)

S3 Appendix. Service readiness score (%) for specific services by province

| 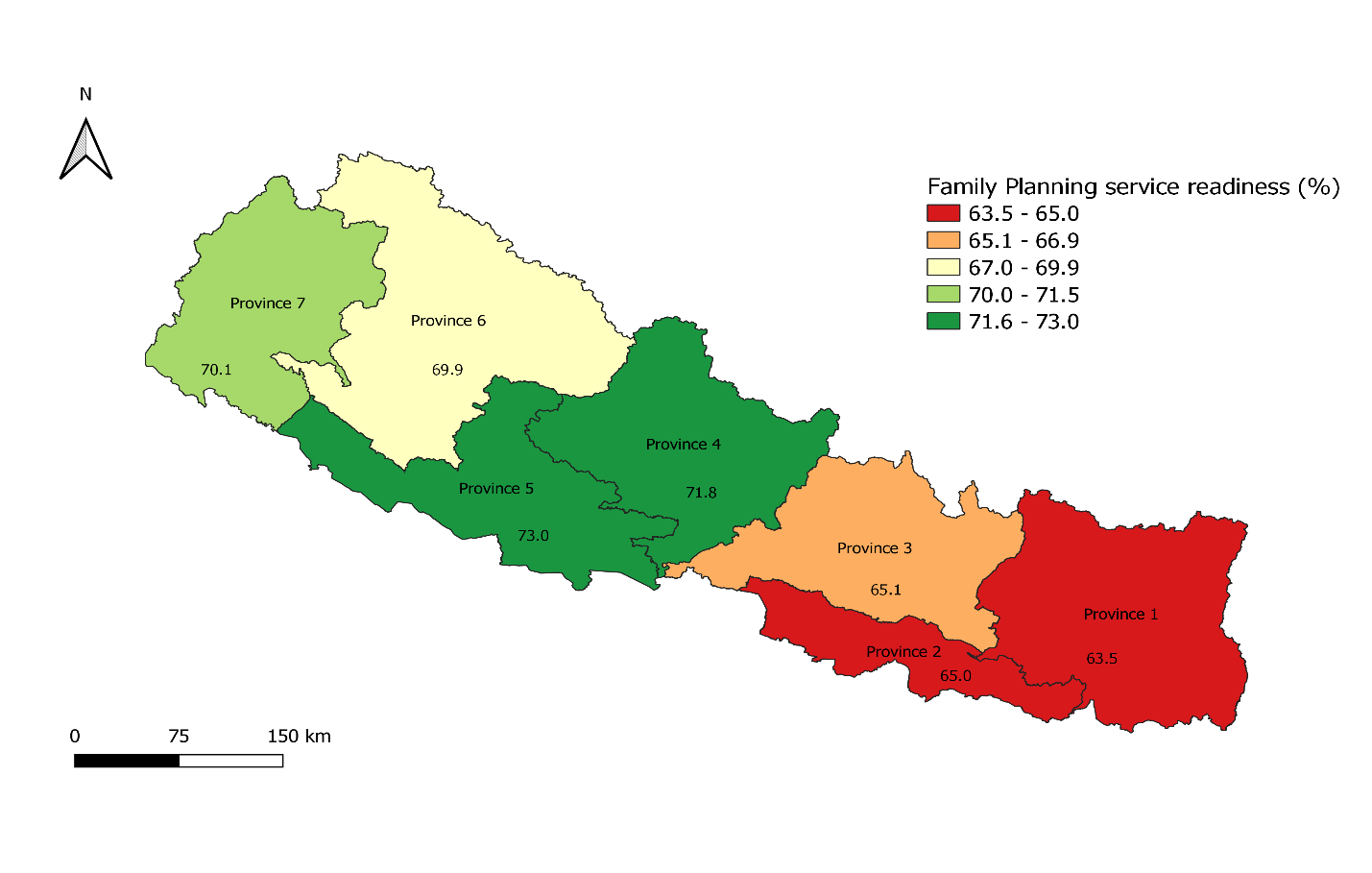  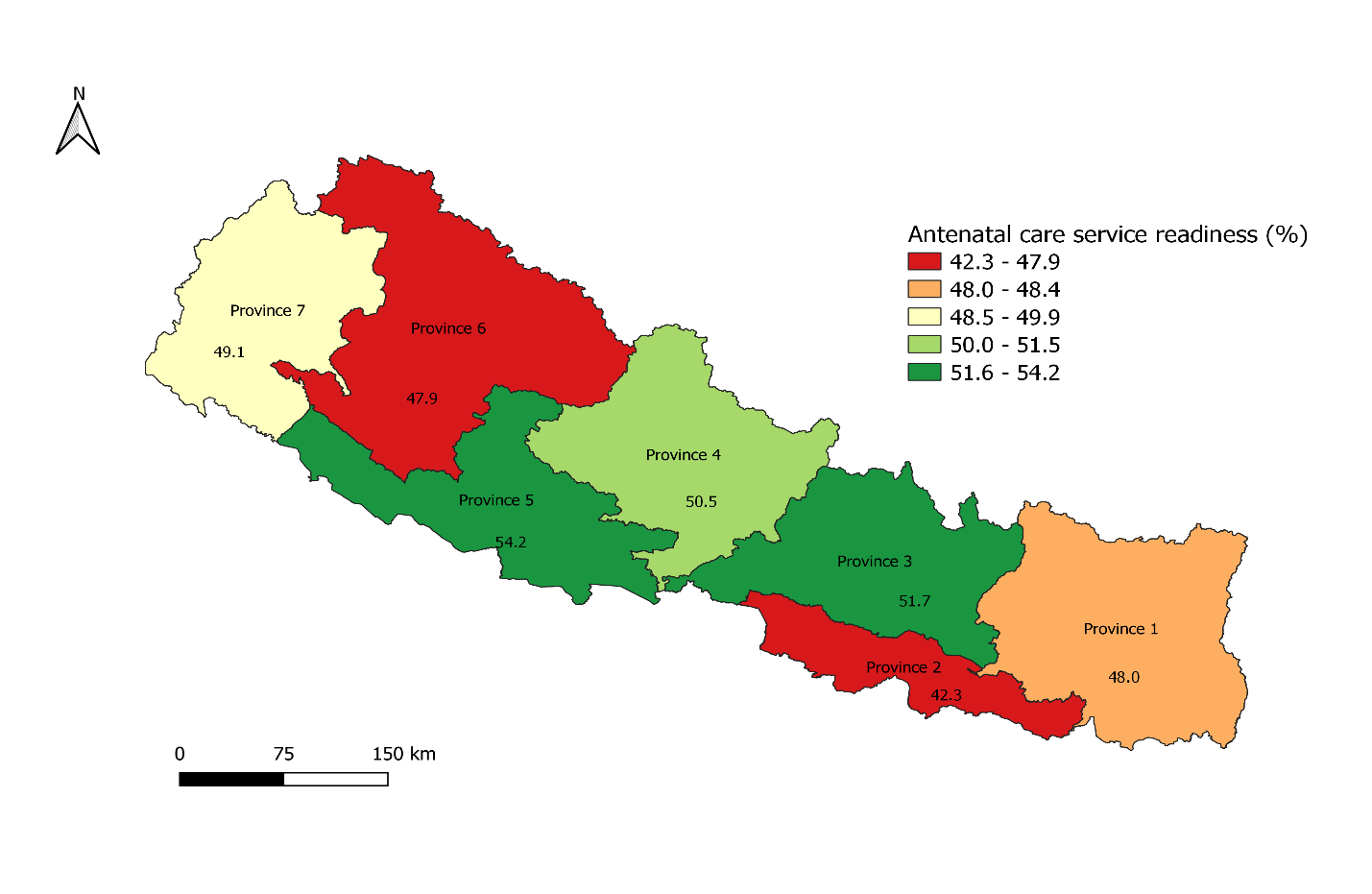  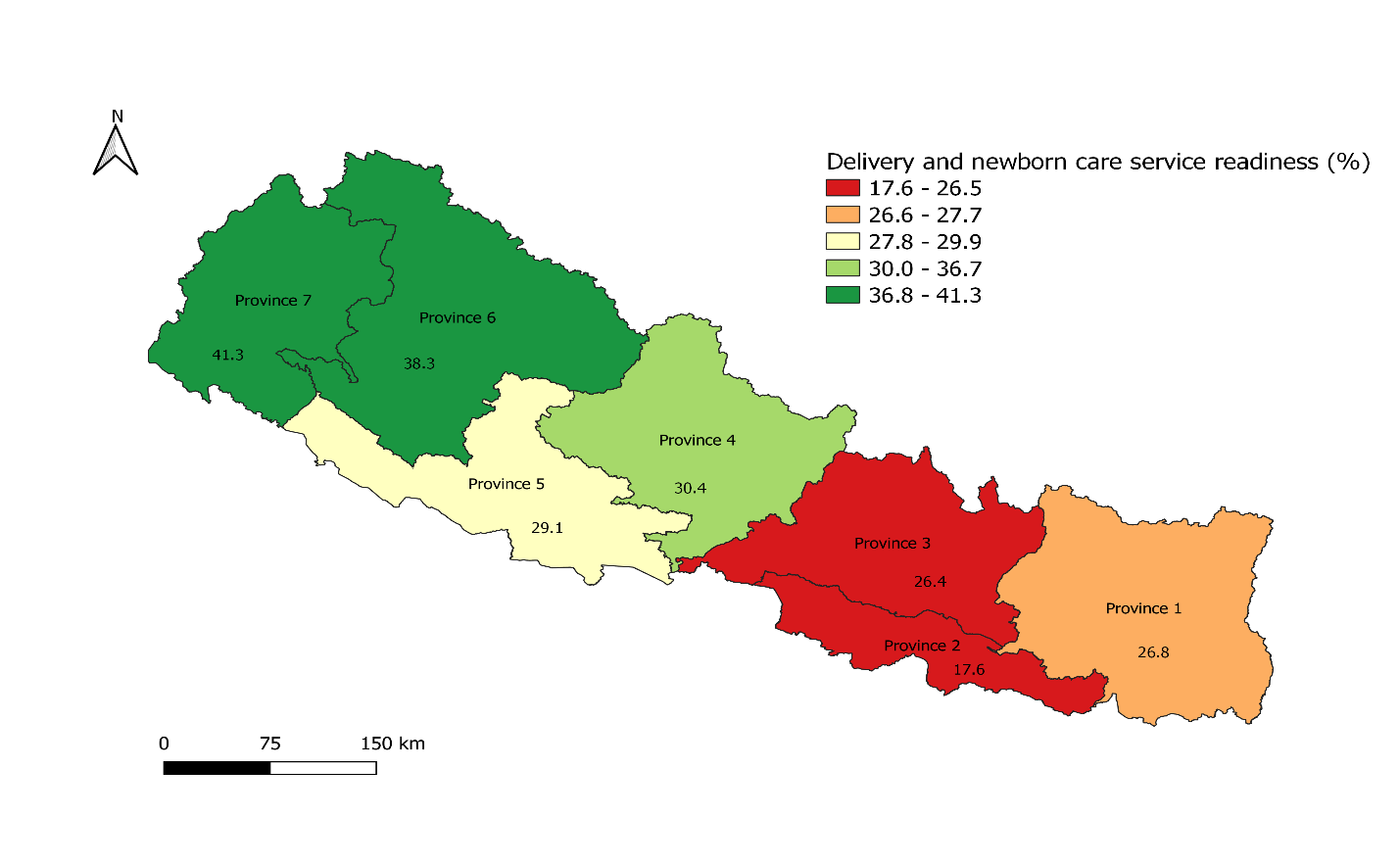  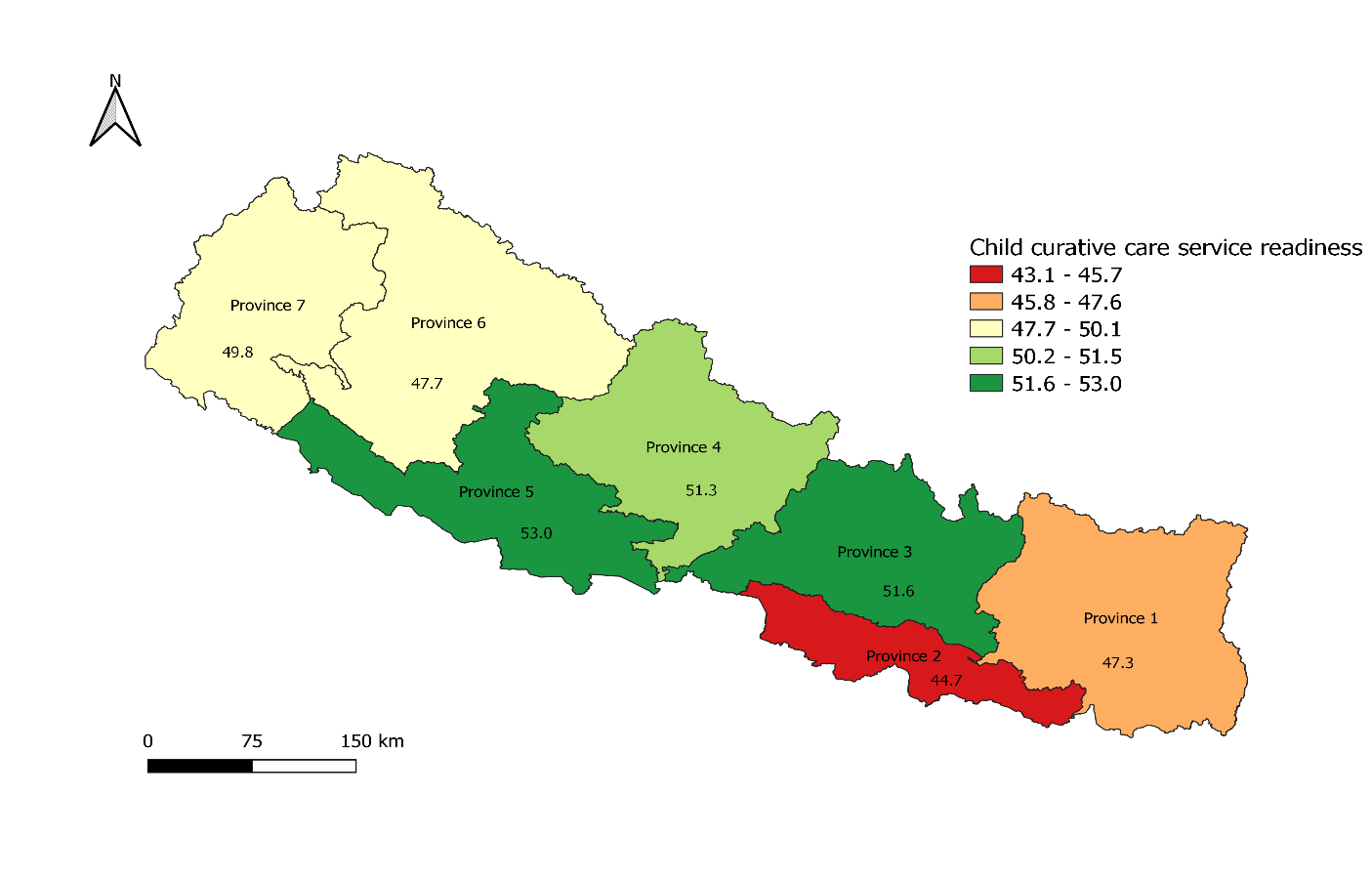 |
| --- |

Supplement: S1 Appendix — (DOCX) [file pone.0264417.s003.docx]
